# PCOLCE Is Potent Prognostic Biomarker and Associates With Immune Infiltration in Gastric Cancer

**DOI:** 10.3389/fmolb.2020.544895

**Published:** 2020-12-18

**Authors:** Aizhai Xiang, Xia Lin, Lvping Xu, Honggang Chen, Jufeng Guo, Fang Zhou

**Affiliations:** ^1^Hangzhou First People’s Hospital, Hangzhou, China; ^2^Department of Breast Surgery, Affiliated Hangzhou First People’s Hospital, Zhejiang University School of Medicine, Hangzhou, China

**Keywords:** PCOLCE, gastric cancer, immune infiltration, prognostic biomarker, prognosis

## Abstract

**Background:**

The exact biological role of PCOLCE was not yet clear and there were few reports study the correlation of PCOLCE gene expression level with the occurrence and development of gastric cancer.

**Methods:**

The expression of PCOLCE was analyzed by performing the Oncomine and Ualcan database. We evaluated the function of PCOLCE on clinical prognosis with the use of Kaplan–Meier plotter database. The relationship between PCOLCE and cancer immune in filtrates was researched by Tumor Immune Estimation Resource (TIMER) site database.

**Results:**

PCOLCE significantly upregulated in gastric cancer patients compared to normal gastric samples. And the increased expression of PCOLCE mRNA was closely linked to shorter overall survival (OS), progress-free survival (PFS) in all gastric cancers. Besides, PCOLCE expression displayed a tight correlation with infiltrating levels of macrophages and dendritic cells (DCs) in gastric cancer. Moreover, PCOLCE expression was positively correlated with diverse immune marker sets in gastric cancer.

**Conclusion:**

All the results above suggested that overexpression of PCOLCE indicated unfavorable prognosis in patients with gastric cancer. PCOLCE was correlated with immune infiltrating levels including those of B cells, CD8 + T cells, CD4 + T cells, macrophages, neutrophils, and DCs in gastric cancer patients. All the findings suggested that PCOLCE could be used as a prognostic biomarker for determining prognosis and immune infiltration in gastric cancer. Additionally, PCOLCE expression potentially contributed to the regulation of monocyte, M2 macrophage, Tfh, CD8 + T cell, TAM, Th1 cell Thus PCOLCE is a potential target for gastric cancer therapy and these preliminary findings require further study to determine whether PCOLCE-targeting reagents might be developed for clinical application in gastric cancer.

## Introduction

Gastric cancer ranks fifth (5.7%) among the most common cancers in the world and third (8.2%) in cancer caused deaths, although its morbidity and mortality have been declining over the past few decades (Ferro et al., 2014; Bray et al., 2018). Gastric cancer remains the major contributor to the global male cancer, second only to lung and liver cancer in disability-adjusted life years (Soerjomataram et al., 2012). Surgical resection combined with perioperative and adjuvant chemotherapy as well as radiotherapy and chemotherapy is an effective method for the treatment of locally advanced gastric cancer (Van Cutsem et al., 2016). However, the prognosis of patients with advanced gastric cancer is always poor, and the average overall survival time is only 10–12 months (Digklia and Wagner, 2016). Gastric cancer is a complex disease involving environmental factors and genetic variation (Tan and Yeoh, 2015). Therefore, understanding the molecular mechanism of gastric cancer is of great significance for finding new therapeutic targets and improving the prognosis of patients.

In oncology, immunotherapy is a hot topic at present, which has shown remarkable results in some cancers, such as renal cancer, melanoma, and non-small cell lung cancer (Larkin et al., 2015; Motzer et al., 2015; Reck et al., 2016). In some cancers, like non-small-cell lung carcinoma (NSCLC) and malignant melanoma, immunotherapy, such as programmed death-1 (PD-1), programmed death ligand-1 (PD-L1) inhibitors and cytotoxic T lymphocyte-associated antigen 4 (CTLA4) showed outstanding antitumor effects (Barbee et al., 2015; Garon et al., 2015). Some reports found that cytotoxic T-lymphocyte associated protein 4 (CTLA-4) and programmed cell death 1 (PD-1) pathways play a key role in Treg cells to impede CD8 T cell growth and upregulate PD-1 expression level in gastric cells, which is correlated with an unfavorable prognosis (Topalian et al., 2012). In clinical trials, Kang et al. (2017) displayed that in the treatment of gastric cancer patients, nivolumab (a fully human IgG4 monoclonal blocking antibody for PD-1) showed good effectiveness and tolerance. And nivolumab could improve the OS of gastric cancer patients (Kang et al., 2017). Moreover, an increasing number of studies have displayed that TILs, such as tumor-infiltrating neutrophils (TINs) and TAMs affect the outcome of gastric cancer treatment (Zhang et al., 2018). Therefore, it is highly urgent to discover the biomarkers of immune interaction with gastric cancer and identify novel immune-related therapeutic targets in gastric cancers.

Procollagen C-protease enhancer protein (PCOLCE) is a secretory glycoprotein, which plays an important role in enhancing the activity of procollagen C-protease and promoting the reconstruction of extracellular matrix (Moali et al., 2005; Vadon-Le Goff et al., 2011; Pulido et al., 2018). PCOLCE binds to C-propeptide of type III procollagen and heparin sulfate through its CUB and NTR domains, respectively, leading to the enhancement of BMP-1 activity and the maturation of collagen precursors (Weiss et al., 2010; Bourhis et al., 2013). It has been reported that the disorder of PCOLCE regulation is involved in the occurrence of a variety of diseases. For example, the upregulation of PCOLCE promotes the metastasis of osteosarcoma (Wang et al., 2019). The mutant PABPN1 binds to PCOLCE and traps it in the nuclear chamber, resulting in ophthalmopharyngeal muscular dystrophy (Raz et al., 2013). However, little is known about the role of PCOLCE in gastric cancer. In this study, we used some bioinformatics network tools [UALCAN, Oncomine, and Kaplan–Meier (KM) plotter database] to evaluate the relationship between the expression of PCOLCE (procollagen C-protease enhancer protein) and the prognosis of patients with gastric cancer. We found that the high expression of PCOLCE is a poor prognostic factor in patients with gastric cancer. At the same time, we used the tumor immune assessment resource (TIMER) to study the correlation between PCOLCE and tumor-infiltrating immune cells in the gastric cancer microenvironment. It was found that PCOLCE was closely related to B cells, CD4 + T cells, CD8 + T cells, neutrophils, macrophages, dendritic cells and other immune infiltration in gastric cancer. Our findings confirm the key role of PCOLCE in gastric cancer and provide the potential relationship and mechanism between PCOLCE and tumor-immune interaction.

In this study, we comprehensively studied the expression of PCOLCE in patients with gastric cancer and its relationship with prognosis in databases such as Oncomine, Ualcan, and Kaplan–Meier plotter. In addition, we analyzed the relationship between PCOLCE and tumor-infiltrating immune cells in different tumor microenvironments through tumor immune assessment resource (TIMER). The results of this study clarify the important role of PCOLCE in gastric cancer and provide the potential relationship and mechanism of PCOLCE and tumor-immune interaction.

## Materials and Methods

### Analysis of the PCOLCE Expression Level Between Cancer Tissue and Corresponding Normal Tissue

Oncomine Database^1^ is a cancer microarray database containing 65 gene expression data sets, including nearly 48 million gene expression measurements from more than 4700 microarray experiments (Rhodes et al., 2004). Using Oncomine (see text footnote 1), we analyzed the expression of PCOLCE in different tumors and corresponding normal tissues.

UALCAN^2^ is an online tool with data from TCGA levels, RNA-seq levels, and clinical data containing 31 cancers. The relative expression of specific genes in different cancer subgroups was analyzed according to different clinicopathological characteristics (Chandrashekar et al., 2017). UALCAN was used to detect the expression of PCOLCE mRNA in gastric cancer and normal gastric tissues.

### Survival Analysis of PCOLCE in Gastric Cancer

The Kaplan–Meier plotter is an online database that uses 10461 cancer samples from GEO to assess the impact of about 54675 genes on survival. At present, breast, liver, ovarian, gastric and lung cancer databases have been provided (Gyorffy et al., 2010, 2012, 2013; Szasz et al., 2016; Menyhart et al., 2018). The database contains a large number of clinical data, such as cancer grade, stage, gender and smoking history. The PCOLCE gene was inputted into the Kaplan–Meier Plotter database^3^, and the high expression group and low expression group were divided into high expression group and low expression group according to the expression level above or below the median level, and the survival curve was obtained. These queues were compared by the Kaplan–Meier survival chart, and the hazard ratio (HR), 95% confidence interval (CI) and logarithmic rank *P*-value were displayed on the web page. A *P*-value < 0.05 was regarded as statistically significant.

### Analysis of the Connection of PCOLCE Expression Level and Immune Infiltrates

Tumor Immune Estimation Resource (TIMER)^4^ is an online database that includes 10,897 samples cover 32 kinds of cancer types from The Cancer Genome Atlas (TCGA) to assess the richness of immune infiltrates and provides a systematic analysis of immune infiltrates across diverse cancer types (Li et al., 2017).

A deconvolution previously published statistical method is used to evaluate the abundance of tumor-infiltrating immune cells (TIICs) from gene expression profiles (Li et al., 2016).

We evaluated the association between the PCOLCE expression and the abundance of immune infiltrates including CD4 + T cells, CD8 + T cells, B cells, macrophages, neutrophils and dendritic cells in gene modules. Moreover, the correlations between the PCOLCE expression and tumor-infiltrating immune cells gene markers were detected via correlation modules. The gene markers of tumor-infiltrating immune cells included markers of CD8 + T cells, monocytes, M1 macrophages, M2 macrophages, B cells, T cells (general), TAMs, follicular helper T (Tfh) cells, T-helper 1 (Th1) cells, T-helper 2 (Th2) cells, T-helper 17 (Th17) cells, Tregs, neutrophils, natural killer (NK)cells, dendritic cells (DCs), and exhausted T cells. These gene markers are referenced in previous studies (Le Pape et al., 2016; Danaher et al., 2017; Siemers et al., 2017). The correlation module which provided the expression scatter plots between a pair of user-defined genes among different type cancer, together with the Spearman’s correlation and the estimated statistical significance. PCOLCE was used for the *y*-axis with gene symbols, and tumor-infiltrating immune cells related marker genes are put in the *x*-axis. The gene expression level was displayed with log2 RSEM.

### Statistical Analysis

The results of the survival curve and Kaplan–Meier graph, UALCAN graph and Oncomine graph are represented by HR and P or COX *P*-values of a log-rank test. An unpaired *T*-test was used for the comparison between two mean values. The correlation of gene expression was evaluated by Spearman’s correlation and statistical significance, and the strength of the correlation was judged according to the following guide for the absolute value: 0.30–0.40 “moderate,” 0.40–0.50 “strong”, Significance was defined at ^∗∗∗^*P* < 0.001, ^∗∗^*P* < 0.01, ^∗^*P* < 0.05.

## Results

### The mRNA Expression Levels of PCOLCE in Different Kinds of Human Cancers

**FIGURE 1:**
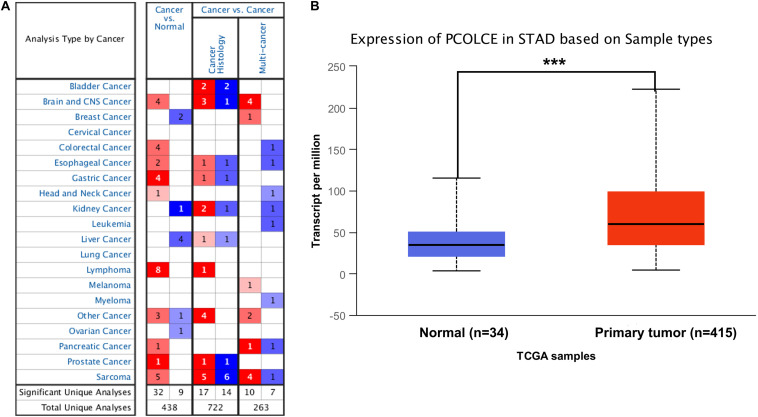
PCOLCE expression levels in different types of human cancers. **(A)** Increased or decreased PCOLCE in data sets of different cancers compared with normal tissues in the Oncomine database. **(B)** The expression of PCOLCE was higher in STAD cancer (stomach adenocarcinoma) compared to normal stomach adenocarcinoma tissues. Data derived from UALCAN database (***P < 0.001).

In order to detect the expression level of PCOLCE in different human tumors and corresponding normal tissues, the mRNA level of PCOLCE was analyzed by using the Oncomine database. The results showed that the expression of PCOLCE in brain and central nervous system, colorectal, esophagus, stomach, head and neck, lymphoma, pancreas, prostate, and sarcoma was higher than that in normal tissues (Figure 1A). In addition, in some data sets, the expression of breast, renal, liver and ovarian cancer is low (Figure 1A).

In order to further evaluate the expression level of PCOLCE in human gastric cancer, we detected the PCOLCE expression using the RNA-seq data of gastric cancer in TCGA with the UALCAN database (see text footnote 2) and the result was shown in Figure 1B. PCOLCE expression was significantly higher in STAD (stomach adenocarcinoma) compared with adjacent normal tissues.

### Prognostic Potential of PCOLCE in Human Cancers

**FIGURE 2:**
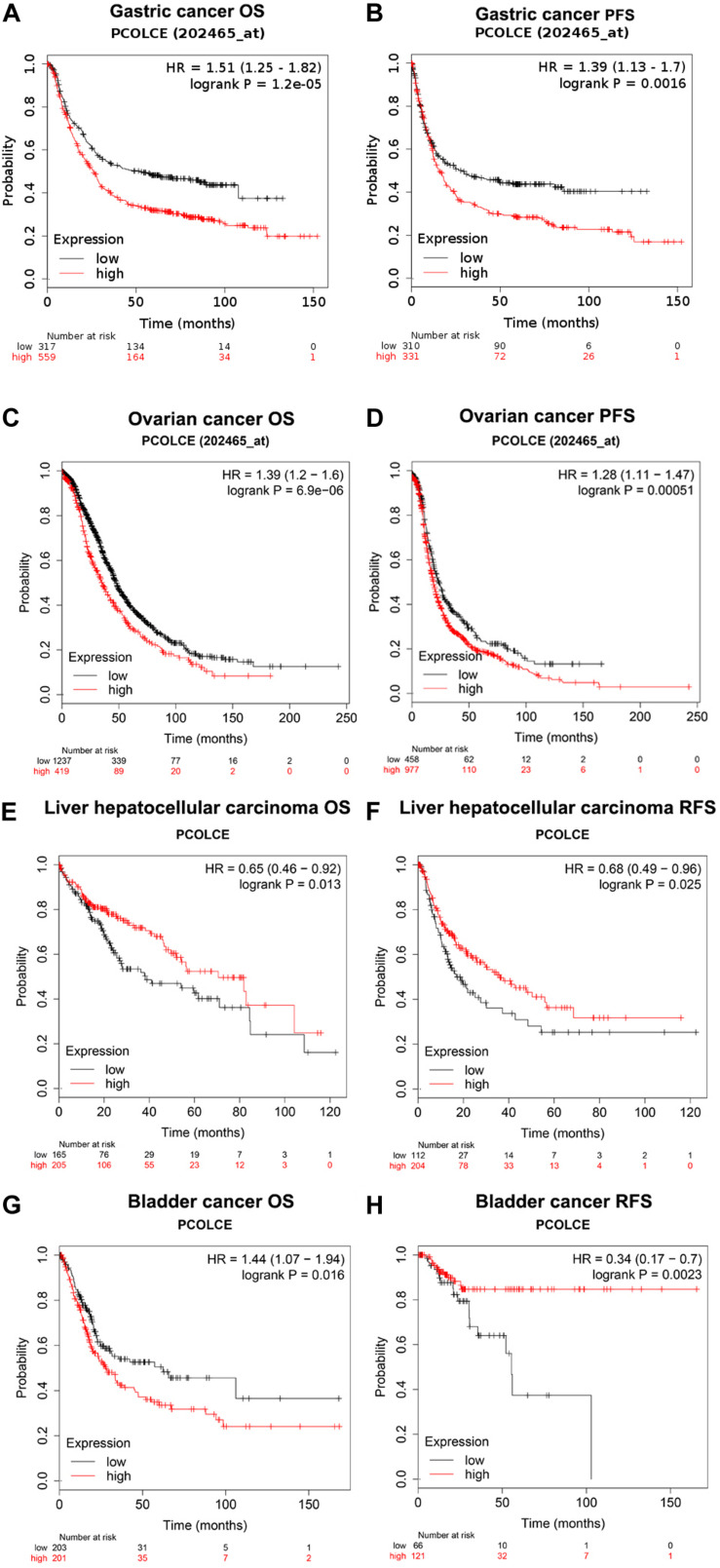
Kaplan–Meier survival curves comparing the high and low expression of PCOLCE in different types of cancer in the Kaplan–Meier plotter databases **(A–H)**. **(A–D)** Survival curves of OS and PFS survival curves of gastric cancer and ovarian cancer. **(E–H)** Survival curves of OS and RFS survival curves of liver hepatocellular carcinoma and bladder cancer. OS, overall survival; DFS, progress-free survival; RFS, relapse-free survival.

We used the Kaplan–Meier plotter database to study whether the expression of PCOLCE was related to the prognosis of cancer patients. It was worth noting that the expression of PCOLCE had a significant impact on the prognosis of gastric cancer, ovarian cancer, hepatocellular carcinoma and bladder cancer (Figures 2A–H). Moreover, in gastric cancer (OS HR = 1.51, 95% CI = 1.25–1.82, *P* = 1.2e-05; PFS HR = 1.39, 95% CI = 1.13–1.70, *P* = 0.0016) and ovarian cancer (OS HR = 1.37, 95% CI = 1.04–1.81, *P* = 0.024; PFS HR = 1.62, 95% CI = 1.14–2.30, *P* = 0.0072), high PCOLCE expression was strongly associated with poorer prognosis (Figures 2A–D). Therefore, it was conceivable that high PCOLCE expression was an independent risk factor and led to a poor prognosis in gastric cancer and ovarian cancer patients.

### Prognosis in Patients With mRNA Expression of PCOLCE and Patient Clinicopathological Features

In order to better understand the potential mechanisms and relationship of PCOLCE expression in gastric cancer, we explored the association of the PCOLCE expression and clinical characteristics in gastric cancer patients with the Kaplan–Meier plotter databases. We collected and evaluated the clinicopathological features of patients with gastric cancer, such as sex, stage, histone grade, lymph node status, metastasis, differentiation, Lauren grade, HER2 status, and so on. In male and female patients, high expression of PCOLCE was associated with worse OS and PFS as well as two types of lauren classification and HER2 statue (*P* < 0.05). Specifically, overexpression of PCOLCE mRNA was linked to worse OS and PFS in stage II to IV of gastric cancer patients (P<0.05) but has little influence on OS or PFS of patients in stage I (OS HR = 1.52, 95% CI = 0.55–4.22, *P* = 0.42; PFS HR = 0.47, 95% CI = 0.15–1.52, *P* = 0.20). Moreover, high expression of PCOLCE mRNA make no sense to the prognosis in negative lymph node status patients (OS HR = 3.07, 95% CI = 0.39–24.37, *P* = 0.26; PFS HR = 1.91, 95% CI = 0.74–4.95, *P* = 0.18) nor patients in metastasis status (OS HR = 1.98, 95% CI = 1.1–3.59, *P* = 0.021; PFS HR = 0.7, 95% CI = 0.39–1.27, *P* = 0.23) (Table 1). In addition, elevated PCOLCE expression indicated poor prognosis in patients with positive status lymph node (OS HR = 1.89, 95% CI = 1.45–2.46, *P* = 1.6e-06; PFS HR = 1.8, 95% CI = 1.4–2.33, *P* = 4.8e-06) and patients without metastasis (OS HR = 1.89, 95% CI = 1.42–2.52, *P* = 1e-05; PFS HR = 1.82, 95% CI = 1.38–2.41, *P* = 1.7e-05). Furthermore, the expression level of PCOLCE had no relationship with the differentiation status of gastric cancer. All the results above suggested that PCOLCE expression level could impact the prognosis in gastric cancer patient with lymph node metastasis and without distance metastasis.

**TABLE 1:** Correlation of PCOLCE mRNA expression and clinical prognosis in gastric cancer with different clinicopathological factors by Kaplan–Meier plotter.

Name	RNA-Seq ID	Clinicopathological features	Overall survival			Progress-free survival		
PCOLCE	202465_at		HR	95% CI	*P*-value	HR	95% CI	*P*-value
		Gender						
		Male	1.48	1.2–1.84	0.00026	1.39	1.09–1.78	0.0071
		Female	1.63	1.12–2.39	0.011	1.65	1.11–2.46	0.013
		Stage						
		I	1.52	0.55–4.22	0.42	0.47	0.15–1.52	0.2
		II	2.05	1.12–3.76	0.018	1.65	0.9–3.03	0.11
		III	1.86	1.36–2.55	9.5e-05	1.93	1.32–2.82	0.00053
		IV	1.75	1.19–2.57	0.004	1.47	1–2.16	0.046
		Lymph node						
		Positive	1.89	1.45–2.46	1.6e-06	1.8	1.4–2.33	4.8e-06
		Negative	3.07	0.39–24.37	0.26	1.91	0.74–4.95	0.18
		Metastasis status						
		Yes	1.98	1.1–3.59	0.021	0.7	0.39–1.27	0.23
		No	1.89	1.42–2.52	1e-05	1.82	1.38–2.41	1.7e-05
		Differentiation status						
		Poor	0.86	0.56–1.32	0.48	1.41	0.76–2.61	0.28
		Moderate	0.53	0.26–1.08	0.077	1.69	0.9–3.18	0.097
		Lauren classification						
		Intestinal	2.36	1.69–3.29	1.7e-07	1.98	1.39–2.83	0.00011
		Diffuse	1.71	1.21–2.41	0.0019	1.7	1.2–2.4	0.0023
		HER2 statue						
		Positive	1.54	1.12–2.12	0.0076	1.67	1.17–2.39	0.0044
		Negative	1.68	1.31–2.15	3.8e-05	1.55	1.2–2.01	0.00074

### PCOLCE Expression Is Correlated With Immune Infiltration Level in Gastric Cancer

**FIGURE 3:**
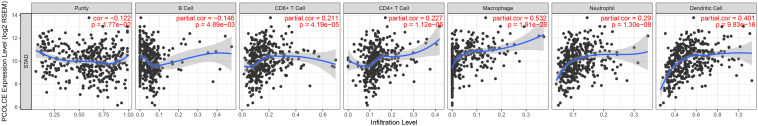
Correlation of PCOLCE expression with immune infiltration level in STAD (stomach adenocarcinoma). PCOLCE expression is significant positive correlations with infiltrating levels of CD8 + T cells, CD4 + T cells, macrophages, neutrophils, and dendritic cells in STAD (stomach adenocarcinoma). While PCOLCE expression has no significant correlations with tumor purity.

A large number of studies have shown that tumor infiltrating lymphocyte (TIL) is an independent predictor of tumor lymph node status and survival (Ohtani, 2007; Azimi et al., 2012). Thus, we evaluated the associations of PCOLCE expression with immune infiltration levels in gastric cancer from the TIMER database. And the results displayed that high PCOLCE expression level had positive correlations with infiltrating levels of CD8 + T cells (*r* = 0.211, *P* = 4.19e-05), CD4 + T cells (*r* = 0.227, *P* = 1.12e-05), macrophages (*r* = 0.532, *P* = 1.91e-28), dendritic cell (*r* = 0.401, *P* = 9.83e-16) and neutrophils (*r* = 0.290, *P* = 1.30e-08) in gastric cancer (Figure 3). While PCOLCE expression had no significant correlations with tumor purity (*r* = −0.122, *P* = 1.77e-02). These findings suggested that PCOLCE might play a specific role in immune infiltration in gastric cancer, especially macrophages and dendritic cell.

### Correlation Exploration Between PCOLCE Expression and Immune Marker Sets

In order to further study the relationship between PCOLCE expression and immune infiltrating cells in gastric cancer, we used TIMER database to detect the immune markers of T cells, CD8 + T cells, B cells, monocytes, neutrophils, NK cells, TAMS, M1 and M2 macrophages, and dendritic cells in gastric cancer. In addition, we also analyzed T cells with different functions, such as Th1 cells, Th2 cells, Tregs, Tfh cells, Th17 cells, and depleted T cells. It is reported that tumor purity is an important factor affecting the results of immune infiltration of tumor samples analyzed by genomic method (Yoshihara et al., 2013). After the correlation adjustment of tumor purity, the results showed that the expression level of PCOLCE in gastric cancer tissues was closely related to most of the immune marker sets of immune cells (Table 2). Notably, we showed CD86, IL10 of monocyte, CD163, MS4A4A, ITGAM, ITGAX of M2 Macrophage, BCL6, STAT5B, HAVCR2 of Tfh showed moderate correlate with PCOLCE expression in gastric cancer (*P* < 0.001; 0.40>Cor value ≥ 0.30). And CSF1R of CD8 + Tcell, CCL2, VSIG4 of M2 Macrophage, NRP1 of Th1, TGFB1 of Treg cell presented strong correction with PCOLCE expression in gastric cancer (*P* < 0.001; Cor value ≥ 0.40).

**TABLE 2:** Correlation analysis between PCOLCE and relate genes and markers of immune cells in TIMER.

Description	Gene markers	STAD					
		None		Purity		Age	
		Cor	*P*	Cor	*P*	Cor	*P*
CD8 + Tcell	CD8A	0.205	0.000	0.177	0.001	0.215	0.000
	CD8B	0.099	0.043	0.080	0.119	0.102	0.039
T cell (general)	CD3D	0.182	0.000	0.136	0.008	0.187	0.000
	CD3E	0.200	0.000	0.158	0.002	0.203	0.000
	CD2	0.204	0.000	0.167	0.001	0.211	0.000
B cell	CD19	0.186	0.000	0.174	0.001	0.183	0.000
	CD79A	0.245	0.000	0.215	0.000	0.254	0.000
Monocyte	CD86	0.366	0.000	0.336	0.000	0.383	0.000
	CSF1R	0.424	0.000	0.404	0.000	0.442	0.000
TAM	CCL2	0.563	0.000	0.538	0.000	0.570	0.000
	CD68	0.213	0.000	0.173	0.001	0.234	0.000
	IL10	0.398	0.000	0.386	0.000	0.413	0.000
M1 Macrophage	NOS2	−0.068	0.165	−0.086	0.094	−0.061	0.223
	IRF5	0.284	0.000	0.281	0.000	0.290	0.000
	PTGS2	0.170	0.000	0.171	0.001	0.187	0.000
M2 Macrophage	CD163	0.352	0.000	0.321	0.000	0.373	0.000
	VSIG4	0.471	0.000	0.452	0.000	0.483	0.000
	MS4A4A	0.422	0.000	0.397	0.000	0.435	0.000
Neutrophils	CEACAM8	−0.091	0.063	−0.089	0.085	−0.104	0.037
	ITGAM	0.419	0.000	0.399	0.000	0.435	0.000
	CCR7	0.224	0.000	0.185	0.000	0.226	0.000
Natural killer cell	KIR2DL1	0.066	0.178	0.054	0.295	0.074	0.139
	KIR2DL3	−0.050	0.311	−0.064	0.210	−0.041	0.411
	KIR2DL4	−0.066	0.177	−0.100	0.052	−0.047	0.341
	KIR3DL1	0.028	0.570	0.018	0.725	0.038	0.450
	KIR3DL2	0.034	0.485	0.012	0.814	0.032	0.521
	KIR3DL3	−0.143	0.003	−0.139	0.007	−0.142	0.004
	KIR2DS4	0.000	0.997	−0.015	0.773	0.012	0.816
Dendritic cell	HLA-DPB1	0.296	0.000	0.251	0.000	0.302	0.000
	HLA-DQB1	0.159	0.001	0.115	0.025	0.168	0.001
	HLA-DRA	0.199	0.000	0.159	0.002	0.213	0.000
	HLA-DPA1	0.226	0.000	0.187	0.000	0.238	0.000
	CD1C	0.283	0.000	0.264	0.000	0.279	0.000
Th1	NRP1	0.506	0.000	0.485	0.000	0.520	0.000
	ITGAX	0.368	0.000	0.335	0.000	0.386	0.000
	TBX21	0.182	0.000	0.156	0.002	0.193	0.000
	STAT1	−0.071	0.148	−0.084	0.101	−0.064	0.197
	IFNG	−0.035	0.483	−0.055	0.288	−0.020	0.686
	TNF	0.180	0.000	0.155	0.002	0.193	0.000
Th2	GATA3	0.308	0.000	0.283	0.000	0.304	0.000
	STAT6	−0.050	0.310	−0.056	0.275	−0.046	0.356
	STAT5A	0.282	0.000	0.271	0.000	0.287	0.000
	IL13	0.159	0.001	0.188	0.000	0.165	0.001
Tfh	BCL6	0.348	0.000	0.316	0.000	0.347	0.000
	IL21	0.007	0.883	−0.009	0.862	0.015	0.757
Th17	STAT3	0.194	0.000	0.174	0.001	0.194	0.000
	IL17A	−0.202	0.000	−0.215	0.000	−0.198	0.000
Treg	FOXP3	0.224	0.000	0.197	0.000	0.235	0.000
	CCR8	0.229	0.000	0.218	0.000	0.247	0.000
	STAT5B	0.312	0.000	0.307	0.000	0.311	0.000
	TGFB1	0.641	0.000	0.624	0.000	0.646	0.000
T cell exhaustion	PDCD1	0.216	0.000	0.195	0.000	0.230	0.000
	CTLA4	0.127	0.009	0.094	0.066	0.139	0.005
	LAG3	0.144	0.003	0.125	0.015	0.162	0.001
	HAVCR2	0.373	0.000	0.354	0.000	0.391	0.000
	GZMB	0.103	0.036	0.066	0.200	0.125	0.012

## Discussion

In this report, we revealed that high PCOLCE expression indicated poor prognosis in patients with gastric cancer. It’s reported that PCOLCE specifically promotes the activity of BMP-1. BMP-1 is a zinc metalloproteinase that removes C-propeptides from the procollagen I, II and III in the extracellular matrix leads to collagen deposition (Moali et al., 2005). It’s well known that increased collagen deposition is the most well-recognized ECM alteration during cancer progression (Lu et al., 2012), indicating that PCOLCE may be involved in the development of cancer metastasis. Moreover, PCOLCE has also been reported may participate in tumor growth based on the interaction network of PCOLCE (Salza et al., 2014). Here, we reported that variations in PCOLCE expression level correlate to prognosis in different types of cancer. The higher expression level of PCOLCE correlated with a poorer prognosis in gastric cancer and ovarian cancer patients. Interestingly, the increased expression of PCOLCE could affect the prognosis of gastric cancer patients with lymph node metastasis, suggesting that the expression of PCOLCE could be used as an index to predict gastric tumor metastasis. In addition, the high expression of PCOLCE also indicated that the prognosis of gastric cancer patients without distant metastasis is poor, suggesting that PCOLCE can be used as a predictor of patients with early gastric cancer.

Furthermore, our analyses display that the level of immune infiltration and different sets of immune markers in gastric cancer were related to the expression level of PCOLCE. The biological function of PCOLCE in tumor is not yet clear, and there are a few studies on the relationship between PCOLCE gene and the occurrence and development of gastric cancer. Therefore, our study provides insight into the potential function of PCOLCE in tumor immunology and its application as a cancer biomarker. In this study, we examined the PCOLCE expression levels in different types of cancers using independent datasets in Oncomine and Ualcan databases. With the Oncomine and Ualcan database, we found that the PCOLCE expressed higher in gastric tumor tissue compared to the normal tissues (Figure 1). With Kaplan–Meier plotter bioinformatics analysis platform (see text footnote 3), we discovered that PCOLCE is an adverse predictor of gastric and ovarian cancer (Figure 2).

Another important discovery of our research is that PCOLCE expression is linked to multiple immune infiltration levels in gastric cancer. Our results demonstrated that there is a moderate to strong positive relationships between PCOLCE expression level and infiltration level of CD8 + T cells (*r* = 0.211, *P* = 4.19e-05), CD4 + T cells (*r* = 0.227, *P* = 1.12e-05), neutrophils (*r* = 0.290, *P* = 1.30e-08), dendritic cell (*r* = 0.401, *P* = 9.83e-16), and macrophages (*r* = 0.532, *P* = 1.91e-28), in gastric cancer (Figure 3).

Moreover, the correlation between the expression of PCOLCE and the marker genes of immune cells instruct the function of PCOLCE in regulating tumor immunology in gastric cancer.

Our results indicated that PCOLCE has the potential to activate monocyte, M2 Macrophage, Tfh, CD8 + T cell, TAM, Th1 cell as the increase in PCOLCE expression positively correlates with the expression of CD86, IL10 of monocyte, CD163, MS4A4A, ITGAM, ITGAX of M2 Macrophage, BCL6, STAT5B, HAVCR2 of Tfh (*P* < 0.001; 0.40>Cor value ≥ 0.30). CSF1R of CD8 + Tcell, CCL2, VSIG4 of TAM, NRP1, TGFB1 of Th1 cell presented significantly correlate with PCOLCE expression in gastric cancer (*P* < 0.001; Cor value ≥ 0.40) (Table 2). These correlations may indicate the potential mechanism of PCOLCE regulating the function of immune cells in gastric cancer. In summary, these findings suggest that PCOLCE plays an important role in the recruitment and regulation of immune infiltrating cells in gastric cancer.

To sum up, the high expression of PCOLCE is associated with poor prognosis and increased immune infiltration of CD8 + T cells, TAM, Th1 cells, monocytes, M2 macrophages, and Tfh in gastric cancer. Therefore, PCOLCE may play an important role in immune cell infiltration in patients with gastric cancer and can be used as a biomarker of prognosis in patients with gastric cancer.

## Data Availability Statement

All datasets presented in this study are included in the article/supplementary material.

## Author Contributions

AX and FZ conceived the project and wrote the manuscript. XL, LX, HC, and JG participated in data analysis, discussion, and language editing. FZ reviewed the manuscript. All authors contributed to the article and approved the submitted version.

## Conflict of Interest

The authors declare that the research was conducted in the absence of any commercial or financial relationships that could be construed as a potential conflict of interest.

## Footnotes

1 www.oncomine.org

2 http://ualcan.path.uab.edu

3 http://kmplot.com/analysis/

4 cistrome.shinyapps.io/timer
